# Localization of the Brainstem GABAergic Neurons Controlling Paradoxical (REM) Sleep

**DOI:** 10.1371/journal.pone.0004272

**Published:** 2009-01-26

**Authors:** Emilie Sapin, Damien Lapray, Anne Bérod, Romain Goutagny, Lucienne Léger, Pascal Ravassard, Olivier Clément, Lucie Hanriot, Patrice Fort, Pierre-Hervé Luppi

**Affiliations:** 1 CNRS, UMR5167, Physiopathologie des réseaux neuronaux du cycle veille-sommeil, Lyon, France; 2 CNRS, FRE3006, Pharmacologie et Imagerie de la neurotransmission sérotoninergique, Université Lyon1, Lyon, France; Pennsylvania State University, United States of America

## Abstract

Paradoxical sleep (PS) is a state characterized by cortical activation, rapid eye movements and muscle atonia. Fifty years after its discovery, the neuronal network responsible for the genesis of PS has been only partially identified. We recently proposed that GABAergic neurons would have a pivotal role in that network. To localize these GABAergic neurons, we combined immunohistochemical detection of Fos with non-radioactive *in situ* hybridization of GAD67 mRNA (GABA synthesis enzyme) in control rats, rats deprived of PS for 72 h and rats allowed to recover after such deprivation. Here we show that GABAergic neurons gating PS (PS-off neurons) are principally located in the ventrolateral periaqueductal gray (vlPAG) and the dorsal part of the deep mesencephalic reticular nucleus immediately ventral to it (dDpMe). Furthermore, iontophoretic application of muscimol for 20 min in this area in head-restrained rats induced a strong and significant increase in PS quantities compared to saline. In addition, we found a large number of GABAergic PS-on neurons in the vlPAG/dDPMe region and the medullary reticular nuclei known to generate muscle atonia during PS. Finally, we showed that PS-on neurons triggering PS localized in the SLD are not GABAergic. Altogether, our results indicate that multiple populations of PS-on GABAergic neurons are distributed in the brainstem while only one population of PS-off GABAergic neurons localized in the vlPAG/dDpMe region exist. From these results, we propose a revised model for PS control in which GABAergic PS-on and PS-off neurons localized in the vlPAG/dDPMe region play leading roles.

## Introduction

Paradoxical sleep (PS) or REM sleep is characterized by cortical activation, rapid eye movements and muscle atonia. We recently showed in the rat that neurons generating PS are specifically active during PS (PS-on or REM-on neurons) and localized in the sublaterodorsal tegmental nucleus (SLD). We further demonstrated that the activation of these putative glutamatergic neurons is gated by GABAergic neurons active during waking (W) and slow wave sleep (SWS) (PS-off or REM-off neurons) [1]. Combining retrograde tracing with cholera toxin B subunit (CTb) and glutamate decarboxylase (GAD) immunostaining, we proposed that these PS-off GABAergic neurons could be localized in three areas namely the ventrolateral periaqueductal gray (vlPAG) and the dorsal part of the deep mesencephalic reticular nucleus (dDpMe), the caudal pontine reticular nucleus (PnC) and/or the SLD itself [2]. In contrast, Maloney et al. [3] combining GAD67 and Fos immunohistochemistry proposed that the PS-off GABAergic neurons are localized in the nucleus pontis oralis (PnO). Finally, Lu et al. [4] showed that part of the SLD PS-on neurons could be GABAergic combining GAD and Fos staining after PS hypersomnia. They proposed that these GABAergic neurons are responsible for PS genesis by means of reciprocal projections with vlPAG/dDpMe PS-off GABAergic neurons. However, the existence of these neurons still remained to be demonstrated.

On the other hand, we showed that the cessation of activity during PS of the PS-off serotonergic neurons of the dorsal raphe nucleus (DRN) and noradrenergic neurons of the locus coeruleus (LC) is due to a tonic GABAergic input [5], [6]. Combining CTb and GAD or CTb and Fos immunostaining after PS hypersomnia, we proposed that the PS-on GABAergic neurons at the origin of this inhibition would be localized in the vlPAG and the dorsal paragigantocellular reticular nucleus (DPGi) [6], [7].

In summary, it is clear from previous work that PS-off and PS-on GABAergic neurons located in the brainstem play a crucial role in PS onset and maintenance. However, the location of these neurons still remained to be determined. To fill this important gap, we decided to localize all brainstem PS-on or PS-off GABAergic neurons. For this goal, we used a highly sensitive and specific double-staining method combining the immunohistochemical detection of Fos with non-radioactive *in situ* hybridization (ISH) of GAD67 mRNA in control rats (PSC), rats deprived of PS for 72 h (PSD) with the inverted flowerpot method and rats allowed to recover for 3 h after such deprivation (PSR). Fos was chosen because even if it is not a perfect marker of activity [8], [9], [10], it is still the best tool to map activated neurons and it was previously used to identify the brainstem neurons responsible for PS genesis [1], [3], [4], [11], [12], [13]. Since we found that the vlPAG/dDpMe region contains the large majority of the Fos-GAD labeled neurons after PS deprivation, we then inactivated them by Mus iontophoretic applications to investigate the possibility that they gate the onset of PS.

## Results

### Neuroanatomy

#### Analysis of the sleep-waking cycle of the animals

During the last 150 min before perfusion, the PSD rats displayed negligible PS amounts (0.03±0.03%; mean±sem) whereas PSC and PSR rats spent respectively 8.80±0,82% and 40.37±4,17% of their time in PS (p<0.05). The PSR animals spent significantly more time in PS compared to the PSC rats (p<0.001). During this period, the PSD animals spent significantly more time in W (66.37±6.38%) than the animals of the two other groups (PSC: 43.40±5.64%, p<0.05, PSR: 24.17±6.71%, p<0.01). In contrast to PS and W, the quantities of SWS did not significantly differ between the three groups of rats (PSC: 47,80±4.85%, PSD: 33.60±6.35%, PSR: 35.53±3.57%).

#### Localization of the Fos-GAD double-stained neurons after PS deprivation

**Figure 1:**
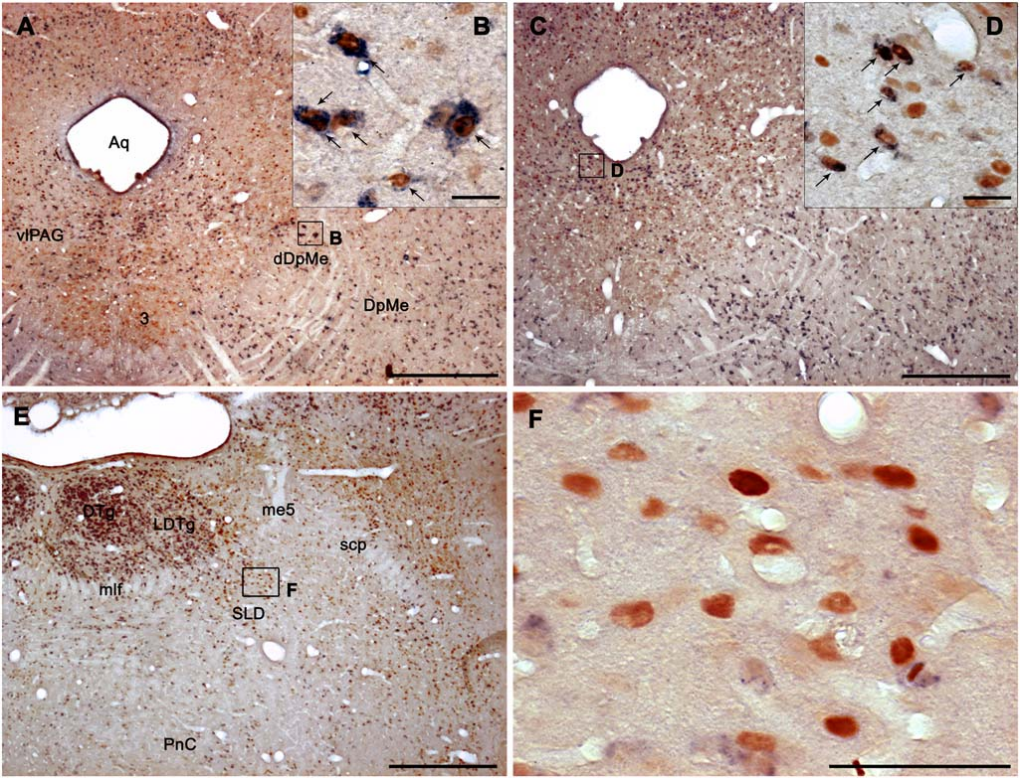
Fos^+^ and GAD67 mRNA^+^ neurons in the vlPAG/DpMe region and the SLD after paradoxical sleep deprivation and hypersomnia. A,C: Low power photomicrographs showing frontal sections double-labeled for Fos and GAD67 at the level of the vlPAG/dDpMe region in PSD (A) and PSR (C) animals. B, D: Enlargements showing several Fos^+^/GAD^+^ neurons (arrows) characterized by a blue diffuse cytoplasmic staining and a brown nuclear staining in the lateral and the medial parts of the vlPAG/dDpMe region in PSD (B) and PSR (D) animals, respectively. E: Low power photomicrograph showing a frontal section double-labeled for Fos and GAD67 at the pontine level in a PSR animal. F: Enlargement of E showing the presence in the SLD of a large number of Fos^+^ and GAD negative labeled cells characterized by a brown nuclear staining. Scale bars: 500 µm for A, C and E; 25 µm for B, D and F. Abbreviations: 3, oculomotor nucleus; Aq, Sylvius aqueduct; dDpMe, dorsal part of the deep mesencephalic nucleus; DpMe, deep mesencephalic nucleus; DRN, dorsal raphe nucleus; DTg, dorsal tegmental nucleus; LDTg, laterodorsal tegmental nucleus; me5, mesencephalic trigeminal tract; mlf, medial longitudinal fasciculus; PnC, pontine reticular nucleus, caudal part; SLD, sublaterodorsal nucleus; vlPAG, ventrolateral periaqueductal gray.

**Figure 2:**
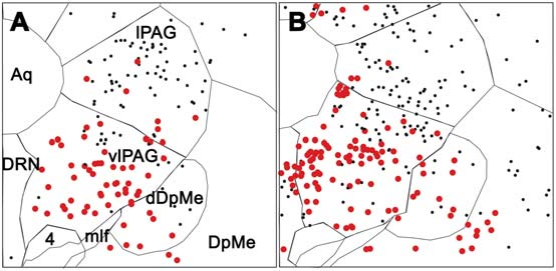
Activated GABAergic neurons in the vlPAG/DpMe region after paradoxical sleep deprivation and hypersomnia (Fos immunohistochemistry combined with GAD67 mRNA *in situ* hybridization). Schematic distribution of Fos^+^ (small black dots) and Fos-GAD (large red dots) neurons in the vlPAG/dDpMe region in PSD (A) and PSR (B) animals (sections −7.40 from Bregma). Double-labeled neurons in the vlPAG are more abundant in the lateral and ventral portions of the vlPAG in the PSD animal and in its medial part in the PSR animal.

The vlPAG contained a very large number of Fos-GAD double-labeled neurons in PSD animals compared to PSC ones (Figures 1A, B, 2A, Table 1 and Figures S1 and S2). The double-labeled neurons constituted 55% of all the Fos-positive (Fos^+^) neurons observed in this structure. The lateral part of the periaqueductal gray (lPAG), dDpMe (Figures 1A, B, 2A, S1, and S2), the parvicellular (PCRt) and gigantocellular reticular nuclei (Gi) contained a substantial number of double-labeled cells (Table 1 and Figures S3 and S4). Importantly, the dDpMe and the PCRt were the only brainstem structures in which the number of Fos-GAD neurons was significantly higher in PSD condition not only compared to PSC but also to PSR condition (Table 1). In addition, the double-labeled neurons localized in the dDpMe and the most adjacent part of the vlPAG were strongly GAD-positive (GAD^+^) and mainly located at the level of the caudal oculomotor nucleus and of the trochlear nucleus (Figure 1A, B). The laterodorsal tegmental nucleus (LDTg) and the caudal part of the DPGi (cDPGi) (Figures S2, S3, S4) contained a small number of double-labeled cells in PSD condition but still significantly above that counted in PSC condition (Table 1). Finally, the percentage of Fos^+^ neurons expressing GAD67 in the cuneiform (CnF), lateral parabrachial nuclei (LPB) and PCRt was similar (34.2% *vs* 34,9% respectively, see above), with a more substantial number of double-labeled neurons in the PCRt compared to the two other nuclei (Table 1 and Figures S2 and S3).

**Table 1:** Number of GABAergic activated neurons in Control, Paradoxical sleep deprived or hypersomniac rats.

	*n*	Fos+Total			Fos+/GAD+			%GAD/Fos		
		PSC	PSD	PSR	PSC	PSD	PSR	PSC	PSD	PSR
**Mesencephalum**										
IP	3	2.3±0.5	10.5±1.7	99.5±22.8***^,###^	1.0±0.4	6.8±0.9	90.0±17.1***^,###^	54.2±20.8	66.5±6.1	92.4±3.4
SNC	2	2.0±2.0	8.8±3.1	16.0±9.5	1.0±1.0	8.3±2.7	14.3±9.7	12.5±12.5	97.1±2.9	72.4±10.7
SNR	2	2.0±1.4	13.5±5.9	30.5±23.7	1.0±0.7	12.5±5.6	28.8±23.2	25.0±14.4	90.4±5.5	86.2±7.7
VTA	4	4.8±2.2	21.8±5.8	59.3±21.4*	3.8±2.5	14.3±5.2	47.5±18.5*	54.2±20.8	57.0±11.5	80.0±4.0
**Periaqueductal gray**										
dlPAG	5	20.3±7.3	50.0±6.0	244.0±67.9**	3.3±1.6	12.0±2.7	73.3±19.9**^,##^	16.5±4.9	23.2±2.2	29.8±3.8
dmPAG	5	10.5±2.4	29.0±6.7	140.5±23.5***^,###^	1.8±0.9	7.5±2.7	29.5±2.4***^,###^	14.0±5.0	24.0±4.7	22.2±2.9
lPAG	5	38.0±19.7	211.5±24.2***	298.0±19.7***^,#^	3.5±1.2	43.8±10.6**	33.5±4.1**	12.3±4.4	19.7±3.8	11.4±1.4
vlPAG	5	37.8±13.8	238.5±23.6***	358.0±67.5**	24.3±8.2	129.8±12.0**	183.3±31.2***	63.2±5.4	54.6±1.9	52.0±4.1
CGPn	3	15.3±4.7	33.5±8.5	60.0±14.8*	5.5±3.9	14.0±3.5	27.8±7.4*	27.7±11.8	49.0±17.3	45.4±7.2
DRN	2	4.0±1.7	20.0±9.4	16.5±3.8	2.0±1.2	5.5±1.5	9.5±2.0**	32.5±19.7	36.8±14.0	59.5±6.5
**Pontomesencephalic tegmenti**										
PPTg	1	1.0±0.7	3.0±0.4	13.8±4.2**^,#^	1.0±0.7	2.8±0.5	11.0±3.0**^,##^	50.0±28.9	91.7±8.3	82.2±6.4
LDTg	2	7.8±3.6	30.5±3.1*	78.8±10.5***^,###^	5.3±2.2	23.3±3.5**	40.8±4.7***^,##^	82.3±10.4	75.5±6.3	52.5±4.1
DTg	2	2.3±0.8	12.8±1.4*	28.5±5.2***^,##^	2.3±0.8	7.0±1.7	23.3±4.5***^,##^	100.0±0.0	54.7±12.3	80.8±2.0
**Mesopontine reticular formation**										
dDpMe	4	8.0±2.9	44.0±2.4***	44.5±3.0***	4.0±1.6	30.8±2.4***	19.3±4.8**^,#^	50.8±10.3	70.3±6.1	42.2±8.7
DpMe	5	17.0±7.0	149.5±51.5	258.0±76.2**	10.0±5.0	75.8±21.7	112.3±38.2*	42.8±14.9	53.4±2.7	38.2±9.1
SLD	2	7.3±3.1	14.5±3.1	55.5±13.0**^,##^	4.8±2.4	7.8±1.1	8.3±1.9	67.0±15.3	57.3±9.0	15.3±3.0
PnO	1	2.5±1.5	24.5±11.1	71.0±23.5**^,##^	1.8±1.4	12.0±5.4	26.0±9.0	31.3±23.7	50.5±7.6	38.8±4.1
PnC	2	10.3±6.5	70.5±18.5	130.5±38.5**	6.0±5.3	35.3±7.0	68.0±20.9**	34.2±17.5	52.9±7.2	53.8±7.4
PnV	1	0.8±0.5	6.3±3.0	14.5±2.8**^,#^	0.3±0.3	5.0±2.5	9.0±2.9	25.0±25.0	58.0±19.6	60.3±10.7
**Dorsolateral pons**										
CnF	1	10.0±3.2	23.3±3.8*	32.8±4.7**	3.0±1.2	7.5±2.9	6.0±1.2	41.5±16.9	34.2±12.7	19.0±4.0
LPB	2	11.0±1.3	89.0±18.7*	111.8±37.1*	3.0±1.0	5.5±2.3	10.8±2.5	26.1±5.8	5.5±1.6	11.0±2.3
LC	1	0.8±0.5	1.5±0.6	1.3±1.3	0.0±0.0	0.0±0.0	1.0±1.0	0.0±0.0	0.0±0.0	20.0±20.0
KF	1	2.5±0.9	18.3±5.6	17.5±5.8	0.5±0.5	0.0±0.0	2.5±0.6*^,##^	16.7±16.7	0.0±0.0	23.3±9.3
MnR	3	1.0±0.7	12.5±3.9	18.5±6.3*	0.8±0.5	1.5±0.6	7.5±1.8**^,##^	41.7±25.0	11.9±5.8	45.0±8.1
**Medullary reticular formation**										
PCRt	6	25.0±12,0	116.8±34.1	70.5±23.9	8.3±4.1	40.3±10.7*	14.8±4.8^#^	30.4±4.9	34.9±2.3	25.3±7.1
Gi	5	6.0±2.0	68.0±14.6*	106.8±27.2**	3.3±1.4	32.3±7.1*	58.3±12.5**	40.6±14.4	46.8±3.4	56.7±7.3
GiA	3	9.3±3.7	37.0±8.4	96.8±14.2***^,##^	3.8±1.0	23.0±3.7	63.0±9.7***^,##^	59.7±16.3	66.3±7.5	64.7±1.9
GiV	2	3.3±1.5	15.0±2.7	59.5±10.9***^,###^	2.3±1.3	4.5±0.5	51.8±9.2***^,###^	46.4±17.6	34.1±8.1	87.8±3.1
rDPGi	1	0.8±0.5	3.8±1.4	25.5±6.2***^,##^	0.5±0.3	3.5±1.5	10.3±4.1	37.5±23.9	66.7±23.6	38.7±10.3
cDPGi	3	5.0±1.2	19.0±5.0	57.3±15.7***^,#^	4.0±1.1	13.5±2.8*	47.5±11.9**^,##^	80.2±8.2	75.0±10.2	86.0±4.3
LPGi	5	24.0±7.4	79.3±12.9	203.5±47.8**^,#^	19.8±5.7	42.8±4.5	145.8±38.8**^,##^	84.6±4.3	55.6±3.7	70.7±4.8
RMg	4	5.0±2.7	29.8±6.6*	47.5±11.4**	4.0±2.0	16.0±1.7	39.3±8.8**^,#^	85.9±8.4	59.9±9.2	83.5±3.0
ROb	2	1.0±0.7	7.5±1.9*	8.3±2.6*	0.5±0.5	0.3±0.3	4.3±1.3**^,##^	16.7±16.7	2.5±2.5	53.3±8.2
RPa	6	8.8±4.9	29.0±2.7**	28.8±3.8**	2.8±1.8	8.0±2.5	19.0±2.1***^,##^	70.0±23.8	26.5±8.2	68.3±8.3

Number (mean±sem) of Fos^+^ and Fos-GAD neurons counted in the brainstem in PSC, PSD and PSR rats on a total of 13 sections at 600 µm intervals through the full rostro-caudal extent of the pons and medulla after Fos immunohistochemistry combined with GAD67 mRNA *in situ* hybridization. The values displayed are an average across 4 animals in each group of the sum of all Fos^+^ neurons (Fos^+^Total) and Fos/GAD67 double-labeled neurons (Fos^+^/GAD^+^) counted on one or several sections (column *n*) depending on the rostrocaudal extent of the structures. %GAD/Fos indicates the percentage of Fos^+^ neurons that are GABAergic. Significance values indicated for individual points are: *P<0.05, **P<0.01 and ***P<0.001 vs PSC; ^#^P<0.05, ^##^P<0.01 and ^###^P<0.001 between PSR and PSD. Abbreviations: cDPGi, caudal part of the dorsal paragigantocellular nucleus; CGPn, central gray of the pons; CnF, cuneiform nucleus; dlPAG, dorsolateral periaqueductal gray; dmPAG, dorsomedial periaqueductal gray; dDpMe, dorsal part of the deep mesencephalic nucleus; DpMe, deep mesencephalic nucleus; DRN, dorsal raphe nucleus; DTg, dorsal tegmental nucleus; Gi, gigantocellular reticular nucleus; GiA, gigantocellular reticular nucleus, alpha part; GiV, gigantocellular reticular nucleus, ventral part; IP, interpeduncular nucleus; KF, Kölliker–Fuse nucleus; LC, locus coeruleus; LDTg, laterodorsal tegmental nucleus; lPAG, lateral periaqueductal gray; LPB, lateral parabrachial nucleus; LPGi, lateral paragigantocellular nucleus; MnR, median raphe nucleus; PCRt, parvicellular reticular nucleus; PnC, pontine reticular nucleus, caudal part; PnO, pontine reticular nucleus, oral part; PnV, pontine reticular nucleus, ventral part; PPTg, pedunculopontine tegmental nucleus; rDPGi, rostral part of the dorsal paragigantocellular nucleus; RMg, raphe magnus nucleus; ROb, raphe obscurus nucleus; RPa, raphe pallidus nucleus; SLD, sublaterodorsal nucleus; SNC, substantia nigra, compact part; SNR, substantia nigra, reticular part; vlPAG, ventrolateral periaqueductal gray; VTA, ventral tegmental area.

#### Localization of the Fos/GAD double-stained neurons after PS recovery

At the mesencephalic level, a large number of Fos-GAD double-labeled neurons was observed in the interpeduncular nucleus (IP) specifically in the PSR group. They composed 92% of the Fos^+^ neurons observed in this nucleus (Table 1 and Figure S1). At this level, a substantial number of Fos^+^ cells, with 80% of them being GAD^+^ were also located in the ventral tegmental area (VTA; Table 1).

All mesencephalic and rostral pontine subdivisions of the periaqueductal gray (PAG) contained a very large number of Fos^+^ neurons in the PSR animals significantly above the number counted in PSC animals. However, the number of Fos^+^ cells was significantly increased compared to the PSD animals only in the dorsolateral (dlPAG) and dorsomedial (dmPAG) subdivisions of the PAG (Table 1). The percentage of double-labeled cells was of 52% in the vlPAG, 30% in the dlPAG and 22% in the dmPAG. Only a small percentage of the Fos^+^ neurons localized in the lPAG were GAD^+^ (11%) (Table 1). Importantly, double-labeled neurons in the vlPAG were more abundant in the medial part of the vlPAG just lateral to the DRN or rostral to it in PSR animals and in the lateral and ventral portions of the vlPAG in PSD animals (Figures 1A–D,2A, B, S1 and S2). We also found that 45% of the Fos^+^ cells in the pontine part of the PAG (CGPn) were double-labeled (Table 1). The DRN contained a small number of Fos/GAD double-labeled cells specifically in the PSR condition (Table 1 and Figure S2). Finally, the DpMe contained significantly more double-labeled cells in the PSR than in the PSC condition (Table 1, Figure 1A, B).

In the pontine tegmentum, the LDTg contained a large number of Fos^+^ cells in PSR condition, a majority of them (52%) being GAD^+^ (Figures 1E, S2, S3 and Table 1). A small number of Fos^+^ neurons mainly GAD^+^ (82%) was observed in the pedunculopontine tegmental nucleus (PPTg; Table 1, Figures S2 and S3). At the same level, the dorsal tegmental nucleus of Gudden (DTg) contained a substantial number of Fos^+^ neurons for their vast majority GAD^+^ (81%; Table 1, Figure S2). Also at pontine level, the SLD contained a substantial number of Fos^+^ cells specifically in the PSR group (Table 1, Figures 1E, F and S3). Importantly, the small number of Fos/GAD double-labeled cells in this nucleus did not significantly vary between the three groups of animals. The PnC but not the PnO contained a large number of Fos-GAD neurons in the PSR condition composing 54% of all the Fos^+^ neurons (Table 1, Figures S2 and S3). In all these pontine structures, the number of Fos-GAD labeled cells was significantly superior compared to PSC and PSD conditions (Table 1). At the same level, the LPB contained a large number of Fos^+^ cells like in PSD animals with only 11% of them being GAD^+^ (Table 1).

**Figure 3:**
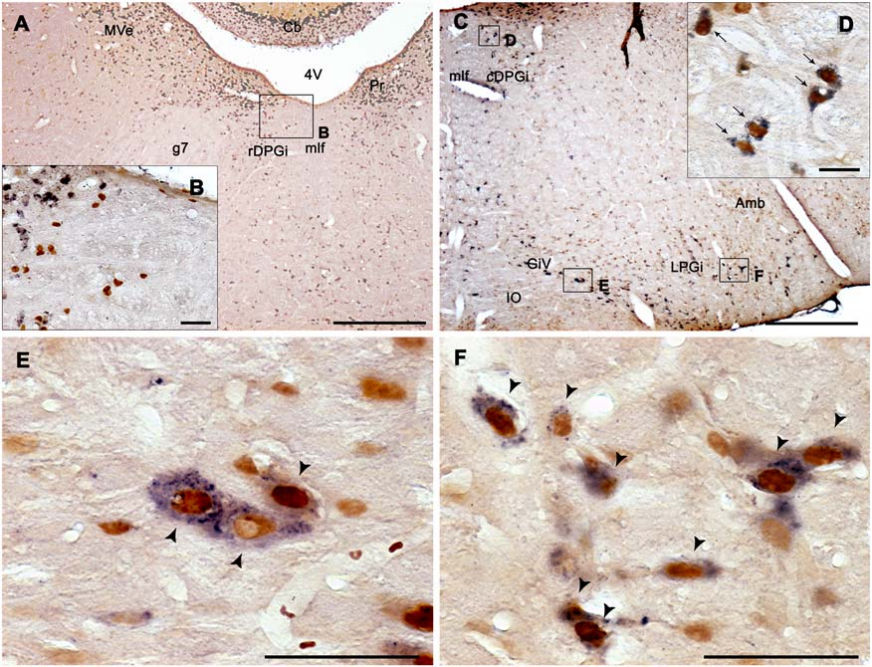
Activated GABAergic neurons at medullary level after paradoxical sleep hypersomnia. Photomicrographs of Fos (brown nuclear staining) and GAD67 mRNA (blue diffuse cytoplasmic staining) double-labeled sections from PSR rats at the medullary level. Low (A) and high (B) power photomicrographs showing the rDPGi. Numerous Fos^+^ and GAD-negative neurons are observed in this structure at high magnification (B). (C–F) Photomicrographs showing low (C) and high magnifications (D–F) of the medullary reticular nuclei in a PSR animal. Note the presence of numerous Fos-GAD cells (black arrows) in the DPGi (D), the GiV (E) and the LPGi (F). Abbreviations: 4 V, 4^th^ ventricle; Amb, ambiguus nucleus; Cb, cerebellum; g7, genu of the facial nerve; mlf, medial longitudinal fasciculus; cDPGi, caudal part of the dorsal paragigantocellular nucleus; GiV, gigantocellular reticular nucleus, ventral part; IO, inferior olive; LPGi, lateral paragigantocellular nucleus; MVe, medial vestibular nucleus; Pr, prepositus nucleus; rDPGi, rostral part of the dorsal paragigantocellular nucleus. Scale bars, 500 µm for A and C; 50 µm for B, E and F and 25 µm for D.

At the medullary level, a large number of Fos^+^ cells, mostly GAD^+^ were localized in the LPGi, DPGi, Gi, the alpha gigantocellular reticular nucleus (GiA), the GiV and to a minor extent the nucleus raphe magnus (RMg) in the PSR condition (Table 1, Figures S3 and S4). Interestingly, in the DPGi, the percentage of double-labeled cells was much higher in its caudal part (cDPGi, 86%) than in its rostral part (rDPGi, 39%) (Table 1, Figures 3A–D and S4). Finally, the obscurus (ROb) and pallidus (RPa) raphe nuclei contained a small number of Fos-GAD cells (respectively 53% and 68% of all the Fos^+^) (Table 1, Figure S4).

### Pharmacology

**Figure 4:**
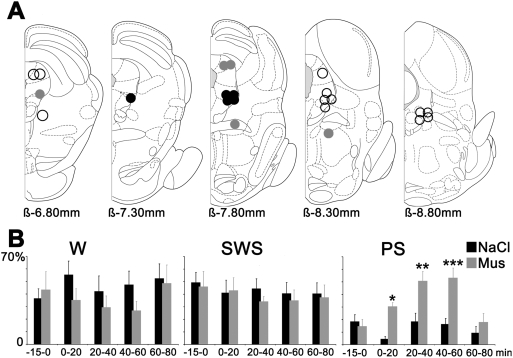
Muscimol injection sites inducing paradoxical sleep hypersomnia. A: Localization on rostro-caudal frontal sections (ß, Bregma) of the Mus injections sites inducing either a PS hypersomnia (black circles), a strong excitation of the animals (grey circles) or no effects on the sleep-wake cycle (empty circles). One circle can represent one or more ejections. B: Percentages of the three vigilance states before and after Mus and NaCl iontophoretic ejections (n = 5) in the vlPAG/dDpMe region. Significance values indicated are: *P<0.05, **P<0.01 and ***P<0.001 vs NaCl.

Following 20 min injections of Mus in the rostral part of the lPAG, the LDTg, the rostral DpMe, and the caudal part of the vlPAG, no change was observed compared to saline injections regarding the sleep-wake cycle (i.e. W, SWS and PS quantities, number of PS episodes and absence of SOREM) (n = 15; Figure 4A). When Mus injections (n = 9) were centered either on the intermediate and caudal part of the lPAG, the ventral part of the DpMe and the rostral part of the vlPAG, a total disappearance of sleep (SWS and PS) concomitant with a strong excitation of rats was observed compared to saline injections (Figure 4A).

**Figure 5:**
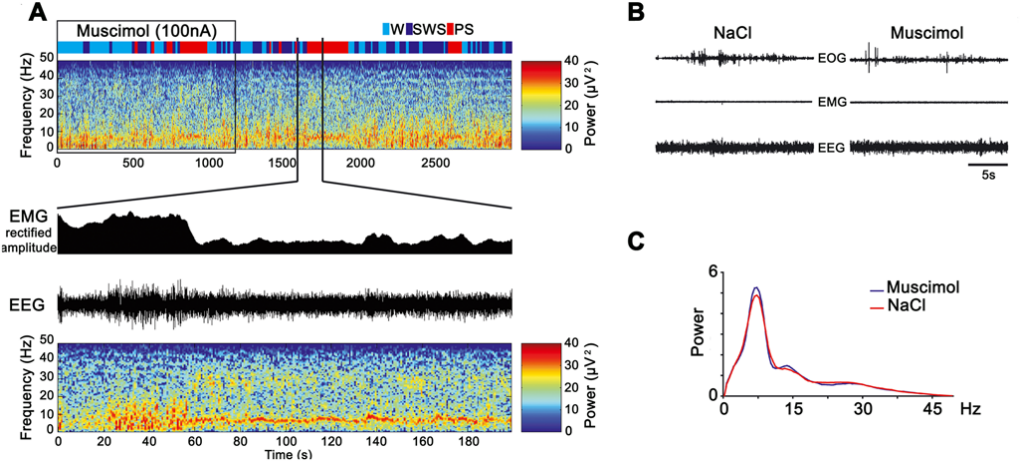
Paradoxical sleep characteristics after muscimol injection in the vlPAG/dDpMe region. A: EEG spectrogram analysis of the 3000 s following one Mus ejection in the vlPAG/dDpMe region. Rectified EMG amplitude, EEG trace and spectrogram analysis are presented for 200 s corresponding to one PS episode induced by the Mus ejection. B: PS episodes induced by Mus ejections share the same properties as the control one, an active EOG, a flat EMG and a dominant theta band frequency activity. C: Average power spectrum analysis for Mus and NaCl ejections in the vlPAG/dDpMe region.

In contrast, following injections of Mus into the vlPAG/dDpMe region (n = 5) (Figures 4 and S5), a dramatic increase in PS quantities (31.9±2.4% of total time) was observed compared to saline injections (9.3±2.9% of total time). PS quantities were increased by 342% compared to NaCl (p = 0.0003) concomitant to a non significant decrease of SWS and W. The dramatic increase of PS was due to an increase in the number of PS episodes (1.3±0.3 for NaCl versus 3.5±0.2 episodes for Mus, an increase of 260%, p = 0.0007) and not of their duration (1.8±0.15 min versus 1.3±0.4 min). The increase of PS started with a mean latency of 7.1±1.3 min and ended 1 hour after the beginning of the Mus injection (Figure 4B). Importantly, 17.3% of the episodes of PS occurring after Mus injections appeared directly after W without a transition by SWS like after saline injections. The episodes of PS occurring after Mus and saline injections were similarly characterized by a cortical EEG desynchronization combined with a muscle atonia and rapid eyes and vibrissae movements (Figure 5A, B and video S1). They also showed the same spectral composition of the EEG (Figure 5C).

## Discussion

In the present study, we demonstrate by means of combined Fos and GAD staining and Mus local applications that large populations of PS-off and PS-on GABAergic neurons are localized in the vlPAG and the adjacent dDpMe and that inactivation of these neurons by Mus induces a large increase in PS quantities. These results suggest that the vlPAG is the most important structure for PS control by mean of its PS-off and PS-on GABAergic neurons. We further showed that SLD PS-on neurons triggering the state of PS [1], [14] are not GABAergic indicating that PS is not generated by a flip-flop switch between PS-on GABAergic neurons localized in the SLD and GABAergic PS-off neurons localized in the vlPAG as recently proposed [4]. Finally, we unexpectedly found that several medullary reticular nuclei contain substantial populations of PS-on GABAergic neurons indicating that GABA in addition to glycine play an important role in sensory and motor inhibition during PS.

In the present study the changes in c-Fos expression are interpreted as reflecting changes in neuronal activity associated with the different experimental conditions. It must be mentioned, nonetheless, that such changes may also reflect changes in other cellular processes that can be stimulated by chemical messengers independent of neuronal discharge, although also dependent on changes in intracellular calcium [15]. In spite of these limitations, c-Fos immunostaining is considered to be a useful tool to map activated neurons [16].

Here we show that the distribution and number of Fos^+^ labeled neurons were similar with our previous publications using the same deprivation recovery method [7], [17] validating our protocol combining ISH of GAD67 and Fos staining. Our results are however largely different with those obtained in the three previous studies reporting the distribution of GAD- and Fos-immunoreactive neurons using nearly the same protocol than us excepting a shorter 48 h PS deprivation [3], [11], [12]. We indeed also observed significantly more Fos-GAD double-labeled cells in the VTA, LDTg, PPTg, CGPn, PnC, RMg, Gi and the GiA after PS recovery compared to control and PSD condition and in the total number of Fos^+^ neurons in the PnO. However, for the latter structure as well as the substantia nigra, median raphe and SLD, we did not observe a change in the number of Fos-GAD neurons in PSR animals compared to the PSD and PSC ones. Further, in contrast to the previous studies, we observed numerous Fos^+^ neurons in the SLD, CGPn, PnC, Gi, GiA, GiV, RMg and the ROb and numerous Fos/GAD labeled neurons in the PnC, cDPGi, GiV and the ROb specifically in PS recovery rats. Finally, we described for the first time the presence of a large number of Fos-GAD double-labeled cells in the vlPAG, dDpMe and PCRt both after PS deprivation and recovery and in the dlPAG, dmPAG, CGPn, IP and DTg only after PS recovery. These discrepancies are likely due to the fact that the Fos antibody used in Maloney et al. [3], [11], [12] studies labeled a large number of cells in basal condition in contrast to the one used in our and the other previous Fos studies likely precluding them to extract the Fos staining specifically induced by the protocol. Further, the development and use in our study of the highly sensitive ISH instead of immunohistochemistry to label GAD-containing neurons likely contributed to a more complete identification of GABAergic neurons.

### Functional role of the GABAergic neurons activated during PS deprivation

**Figure 6:**
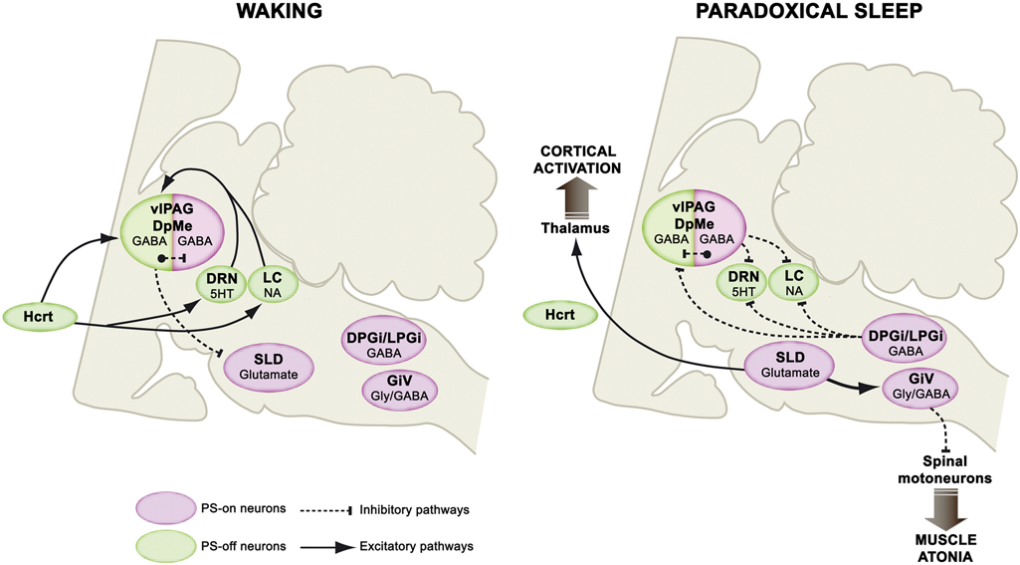
Model of the network responsible for PS onset and maintenance. The SLD contains glutamatergic neurons responsible for the onset and maintenance of PS. They induce muscle atonia and sensory inhibition via direct excitatory projections to the glycinergic/GABAergic neurons localized in the medullary ventral reticular nuclei (GiA, GiV, RMg) and EEG activation via direct intralaminar thalamic projections. During W and SWS, the SLD neurons are tonically inhibited by GABAergic PS-off neurons localized in the vlPAG/dDpMe region. At the onset and during PS these PS-off neurons are tonically inhibited by the co-localized GABAergic PS-on neurons as well as those of the DPGi and LPGi. These GABAergic PS-on neurons are also responsible for the inhibition of locus coeruleus (LC) noradrenergic and dorsal raphe (DRN) serotonergic neurons during PS. In normal condition, the onset of PS is not possible directly from W because the wake-active hypothalamic hypocretinergic neurons and brainstem monoaminergic neurons tonically excite the vlPAG/dDpMe PS-off GABAergic neurons. The decrease or cessation of activity of these wake-active neurons during SWS weaken the activity of vlPAG/dDpMe PS-off GABAergic neurons inducing a desinhibition of the co-localized GABAergic PS-on neurons and by this way PS.

We showed for the first time that PS deprivation activates a large number of GABAergic neurons only in the vlPAG/dDpMe region. The PCRt was the only other brainstem structure containing a substantial number of double-labeled neurons after PS deprivation. Furthermore, we demonstrated that unilateral Mus iontophoretic injections specifically in the vlPAG/dDpMe region induced a strong increase in PS quantities. These pharmacological data are coherent with what has been shown in guinea pigs [18], cats [19], [20] and after chemical lesions in rats [4]. These results combined with our previous pharmacological and neuroanatomical results [1], [2], [21] indicate that PS-off GABAergic neurons localized in the vlPAG/dDpMe region inhibit PS onset during W and SWS by means of a tonic inhibitory input to the SLD PS-on neurons. In addition, we found that 17% of the PS episodes induced by Mus injections appeared directly after W like the SOREMs of narcolepsy [22]. These results combined with the previous findings of hypocretin fibers and receptors in the vlPAG/dDpMe region [4], [23], [24] support the hypothesis that the SOREM and the cataplexy seen in narcoleptics could be due to the lack of an excitatory hypocretin input to the PS-off GABAergic neurons of the vlPAG/dDpMe region (Figure 6).

### Functional role of the GABAergic neurons activated during PS hypersomnia

After PS recovery, a large number of Fos^+^/GAD^+^ neurons was observed in the vlPAG and to a minor extent the dlPAG, dmPAG and the lPAG. A large number of Fos-GAD labeled cells was also seen in the vlPAG after PS deprivation (see above) indicating that this structure contains GABAergic neurons activated during PS deprivation and PS rebound. It is likely that these GABAergic neurons form two distinct populations of PS-off and PS-on neurons, respectively. Indeed, the majority of the double-labeled neurons after PS deprivation were localized in the lateral and ventral portion of the vlPAG whereas they were more numerous in the medial part of the vlPAG just lateral to the DRN or rostral to it after PS recovery. Further, we previously showed that a substantial number of Fos^+^ vlPAG neurons labeled after PS recovery but not after PS deprivation project to the LC [7].

In our previous studies [5], [6], we demonstrated that GAD-IR neurons of the vlPAG project to the DRN and that the cessation of activity of DRN serotonergic and LC noradrenergic neurons during PS is reversed by the iontophoretic application of Bic, a GABA_A_ antagonist [5], [6]. Based on these previous and the present results, we propose that the PS-on GABAergic neurons localized in the vlPAG inhibit during PS the DRN serotonergic and LC noradrenergic neurons and the co-distributed vlPAG/dDpMe GABAergic PS-off neurons inducing by this way the desinhibition of the SLD PS-on neurons. Reciprocally, during W and SWS the vlPAG/dDpMe GABAergic PS-off neurons would inhibit PS by their projections to the co-distributed GABAergic PS-on neurons and the SLD glutamatergic PS-on neurons (Figure 6). However, since Mus injection in the vlPAG/dDpMe region inhibits at the same time the PS-off and PS-on GABAergic neurons localized in this area and nevertheless induces an increase of PS quantities, it might be concluded that the activation of the vlPAG PS-on GABAergic neurons is not essential to obtain PS. It might also indicate that the main function of these neurons is to inhibit the co-localized GABAergic PS-off neurons during PS whereas their role in inhibiting the monoaminergic neurons is not essential. Indeed PS-on GABAergic neurons of the DPGi also likeky contribute to this inhibition (see below and [21]). Our results only partly fit with the recent model of Lu et al. [4] who proposed that the genesis of PS is due to a GABAergic inhibitory reciprocal interaction between PS-on GABAergic neurons localized in the SLD and GABAergic PS-off neurons from the vlPAG/dDpMe region. Indeed, our findings that the small number of Fos-GAD neurons in the SLD did not change in PSC, PSD and PSR animals whereas the total number of Fos^+^ neurons significantly increased in the PSR condition are not fitting with this model. It is more likely that the Fos^+^ neurons observed in the SLD specifically after PS recovery are glutamatergic. Indeed, Lu et al. [4] also reported the presence of vGlut2 containing neurons in the SLD and we have shown that when SLD neurons are desinhibited by Bic, they excite glycinergic neurons of the RMg, GiA and Giv and also the intralaminar thalamic relay neurons [1].

Specifically after PS recovery, five medullary nuclei, the GiA, GiV, RMg, DPGi and LPGi contained a large number of Fos^+^ neurons, mostly GAD-positive. It has been previously shown that the GiA, GiV and RMg display a large contingent of glycinergic neurons [25] projecting directly to spinal motoneurons [26]. We also showed that glycinergic neurons from these nuclei express Fos after a pharmacological induction of PS [1], [13]. In addition, it has been shown that strychnine (a glycine antagonist) but not Bic iontophoresis on motoneurons reverse their hyperpolarization during PS indicating that the premotoneurons responsible use glycine and not GABA to hyperpolarize them during PS [27]. Nevertheless, it was more recently shown that both the release of glycine and GABA increase in the spinal cord and the hypoglossal motor nucleus during the atonia produced by a cholinergic stimulation of the peri-LCα (cat equivalent of the SLD)[28]. Altogether, the present and previous results suggest that the GiV, GiA and RMg PS-on neurons responsible for sensory and motor inhibition during PS co-contain glycine and GABA.

Besides, we previously showed that Fos^+^ cells localized in the DPGi after PS recovery were retrogradely-labeled after CTb injection in the LC [7]. We further showed that the DPGi project to the vlPAG/dDpMe region and to a minor extent to the DRN and the hypothalamic region containing the hypocretin neurons [29]. GABAergic PS-on neurons of the DPGi identified in the present study could therefore in cooperation with those of the vlPAG/dDpMe region participate in the inhibition of the monoaminergic and the vlPAG/dDpMe PS-off GABAergic neurons at the onset and during PS (Figure 6). Finally, we surprisingly found that the LPGi was the second structure after the PAG containing the largest number of Fos-GAD neurons after PS recovery. Since a small subset of Fos^+^ cells localized in the LPGi after PS recovery project to the LC [7], we propose that LPGi PS-on GABAergic neuron contribute to the inhibition of the PS-off neurons during PS.

### Conclusions: a revised model for PS control

The results described in the present study provide substantial progress in the identification of the network responsible for PS control. From these results and previous ones, we propose a revised model for PS control (Figure 6). This model provides a new framework for the identification of the mechanisms controlling PS onset and maintenance and a better understanding of sleep disorders such as narcolepsy and REM sleep behavior disorder.

## Materials and Methods

All experiments were conducted in accordance to the French and European Community guidelines for the use of animals in research. Sprague–Dawley rats were housed individually and placed under a constant light/dark cycle (light on from 7:00 AM to 7:00 PM). The room temperature was maintained at 21±1°C and standard rodent food and water were available *ad libitum* throughout the experiment. The protocols were approved by the institutional animal care and use committee of the University of Lyon 1 (protocols BH 2006-10, BH 2006-09 and BH 2006-07).

### Neuroanatomy

#### Animal and surgery

Briefly [7], [17], [30], twelve male Sprague-Dawley rats (200–230 g, Charles River, France) were implanted with electroencephalogram (EEG) and electromyogram (EMG) electrodes under chloral hydrate anesthesia (400 mg/kg, i.p.). Four stainless steel screws were fixed in the occipital, parietal and frontal parts of the skull and two wire electrodes inserted into the neck muscles to monitor the EEG and the EMG, respectively. All leads were connected to a miniature plug that was cemented to the skull. After surgery, rats were placed in a Plexiglas jar covered with woodchips for the duration of the experiment. Animals were allowed 5 days of recovery from surgery in the animal room before being placed in a recording chamber for 4 days, time necessary for a good habituation to the recording conditions (recording chamber and cable).

#### Polygraphic recordings

Rats were connected to a cable attached to a rotative connector to allow free movement of the animal within the jar. EEG and EMG recordings were collected on a computer via a CED interface using the Spike 2 software (Cambridge Electronic Design, Cambridge, UK).

#### Paradoxical sleep deprivation procedure

PS deprivation was performed using the flower pot technique that has been previously shown to induce a fairly selective deprivation of PS in rats [3], [7], [11], [17], [30], [31]. This procedure does not cause significant changes in adrenal gland weights [31] and plasma corticosterone levels [32], two physiological parameters used to estimate the stress level in animals. Moreover, EEG spectral characteristics of W, SWS and PS states are not modified during the PS deprivation and the recovery period compared to baseline recordings [11].

Rats were divided in 3 groups: control (PSC), deprived of PS for ∼72 h (PSD) and rats allowed to recover for ∼3 h after such deprivation (PSR) (n = 4 for each group). The PSC animals remained on a bed of woodchips in the recording room throughout the experiment. On the first day (9:00 AM) and after 48 h of baseline, PSD and PSR rats were placed on a platform surrounded by 2 cm of warm water for 72 h. The platform was just large enough (6.5 cm in diameter) to hold the animal. In this situation, the animal could engage in SWS but not PS, because of the loss of muscle tonus occurring at PS onset. Everyday in the morning (between 9:00 and 10:00 A.M.), in order to clean their jar, rats were removed from their platform and placed in a Plexiglas jar. The last day, PSC animals were first anesthetized for perfusion (at ∼1:00 P.M.). PSD animals were secondly anesthetized for perfusion (at ∼1:00 P.M.), after 75 h of PS deprivation. The PSR animals were also removed from their cages (10:00 A.M.) and were returned to a dry bed of woodchips in their recording jars to allow PS recovery. PSR rats were anesthetized for perfusion (at ∼1:00) exactly 150 min after the first PS episode occurring generally after ∼30 min of exploration and grooming.

#### Perfusion and fixation

Under profound barbiturate anesthesia (pentobarbital, Ceva santé animale, 150 mg/kg, i.p.), rats were transcardially perfused with Ringer's lactate solution containing 0.1% heparin, followed by 500 ml of a fixative solution composed of 4% paraformaldehyde in 0.1 M phosphate buffer (PB, pH 7.4). The brains were then stored at 4°C for 1 night in the fixative solution and for at least 3 days in 30% sucrose in 0.1 M PB. After that, they were rapidly frozen with CO_2_ gas and 30 µm-thick coronal sections were cut on a cryostat. The free-floating sections were collected and stored at −20°C in a RNase free cryoprotectant solution (0.05% DEPC, 20% glycerol, 30% ethylen glycol in 50 mM PB, pH 7.4).

#### Fos immunohistochemistry combined with GAD67 mRNA in situ hybridization

As described before [33], the recombinant plasmid containing the GAD67 cDNA was linearised using EcoRV and transcribed using SP6 RNA polymerase and a nonradioactive RNA labeling kit (Roche Diagnostic, Mannheim, Germany) for the antisense probe. The brain sections were successively incubated in a rabbit antiserum to Fos (1∶4000; Ab-5; Oncogene, CA, USA) in 10 mM PB containing 0.9% NaCl and 0.3% Triton-100× (PBST) for 18 h at room temperature; a biotinylated goat antirabbit IgG solution (1∶1000; Vector Laboratories, Burlingame, CA, USA); and an ABC-HRP solution (1∶1000; Elite kit, Vector Laboratories) both for 90 min at room temperature. Finally, the sections were immersed in a 0.05 M Tris-HCl buffer (pH 7.6) containing 0.025% 3.3′-diaminobenzidine-4 HCl (DAB, Sigma-Aldrich, St. Louis, MO, USA) and 0.003% H_2_O_2_. Three washes of 10 min in PBST were performed between each step. The sections were then rinsed two times in PBST containing 10 mM dithio-threitol (DTT, Sigma-Aldrich) for 10 min at room temperature and then in standard saline citrate solution (SSC 2×) for 10 min also at room temperature. All the buffers excepting DTT contained 0.2% of RNase inhibitor (solution Protect RNA RNase inhibitor, Sigma-Aldrich). After these rinses, the sections were placed for 18 h at 65°C in a hybridization buffer consisting of NaCl (150 mM), Tris-HCl (8 mM), Tris-Base (1 mM), NaH_2_PO_4_ (6 mM), Na_2_HPO (5 mM), EDTA (5 mM), formamide (50%), dextran sulphate (10%), yeast tRNA (Sigma type III, 1 mg/ml, Sigma-Aldrich), ficoll (0.02%), polyvinylpyrrolidone (0.02%) containing 0.5 µg/ml of the digoxigenin-labeled probe. After hybridization, the sections were washed in SSC 1×, 50% formamide, 0.1% Tween-20 at 55°C twice for 20 min and treated with 10 µg/ml RNase A (USB, Cleveland, OH, USA) in Tris 10 mM (pH 8.0) containing 1 mM EDTA and 500 mM NaCl for 15 min at 37°C. They were then rinsed 3×10 min at room temperature with PBST. For immunohistological detection of digoxigenin (DIG), the sections were incubated overnight at room temperature with anti-DIG conjugated to alkaline phosphatase (Roche Diagnostic) diluted 1∶2000 in PBST containing 0.2% blocking agent (Roche Diagnostic). After incubation, the sections were washed in PBST twice for 10 min and then in a buffer containing 100 mM Tris (pH 9.5), 100 mM NaCl and 50 mM MgCl_2_. The sections were developed at 37°C for ∼4 h in the same buffer with nitroblue tetrazolium (NBT) and 5-bromo-4-chloro-3-indolyl-phosphate (BCIP) diluted 1/50 from the stock solution (Roche Diagnostic). Finally, the sections were mounted on glass slides, dried and coverslipped with Vectamount (Vector Laboratories). Controls in the absence of primary antibodies (anti-Fos and anti-DIG) and with the sense probe (GAD67 cDNA linearised using HindIII and transcribed using T7 RNA polymerase) were run to ensure the absence of nonspecific labeling. As specified by the supplier, the Fos antiserum was made against a synthetic peptide corresponding to the N-terminal part (residues 4–17) of human Fos. This part of the protein displays 100% homology between human and rat (van Straaten et al., 1983; Curran et al., 1987) and no homology with Fos-related antigens such as Fos B, Jun B, Fra-1 and Fra-2 (Blast 2 sequences, NCBI). In agreement with previous studies, very few immunoreactive (IR) nuclei were observed in the rat brain with this Fos antiserum following perfusion during daylight (Semba et al., 2001; Ro et al., 2003; Gong et al., 2004). Brains from sets of PSC, PSD, PSR animals that were run together experimentally were processed together for immunohistochemistry and ISH.

#### Analysis of sleep-wake state data

Vigilance states were discriminated with the aid of EEG and EMG data as previously described (Maloney et al., 2002). For 3 rats per condition, the last 3 h of EEG/EMG recordings before perfusion were analyzed in order to determine quantities, number and duration of periods of W, SWS and PS.

#### Analysis of double labeling

Because the distributions of Fos+ and Fos-GAD neurons were similar on both brain sides, only hemi-sections were analyzed. Drawings of double-labeled sections were made with an Axioscope microscope (Zeiss, Germany) equipped with a motorized X–Y-sensitive stage and a video camera connected to a computerized image analysis system (Mercator; ExploraNova, La Rochelle, France). Single- and double-labeled neurons were plotted on sections taken at 600 µm intervals (13 sections between AP −5.60 and −12.80 from Bregma). The number of Fos^+^ and Fos-GAD plotted neurons per structure was automatically counted and exported using Mercator (ExploraNova). When a structure was present on several sections the neurons counted on all sections were summed. The counts were not performed in the superior and inferior colliculus. The atlas of Paxinos & Watson [34] was used as a reference for all structures except the SLD which was named and delineated according to Swanson [35].

#### Statistical analysis

Analyses of variance (ANOVA) tests were performed on the different vigilance states and the number of labeled neurons for each structure across experimental conditions (PSC, PSD and PSR). Post-hoc PLSD Fisher tests were used to identify significant pairwise differences (PSR or PSD vs PSC; PSR vs PSD). All statistics were performed using Statistica 6.0.

### Pharmacology

#### The head-restrained rat method

The method has been previously described in detail [1], [5], [6]. Briefly, male Sprague–Dawley rats (280–320 g, n = 7; Charles River, France) were anaesthetized with chloral hydrate (400 mg/kg, i.p) and mounted conventionally in a stereotaxic frame (David Kopf, CA, USA). The skull was placed at a 15° angle (nose tilted down) and an additional angle of 15° was applied to the micromanipulator (leading to a total angle of 30°) to avoid the transverse sinus overlying the vlPAG/dDpMe region during the subsequent electrode penetrations. Five electrodes were fixed in the skull bilaterally above the frontal (Bregma +4 mm AP and ±2 mm L) and parietal (Bregma −3 mm AP and ±3 mm L) cortices, and unilaterally above occipital cortex (Bregma −9 mm AP and −3 mm L) to monitor the EEG. Two wire electrodes were inserted into the neck muscles and two electrodes were inserted behind each ocular globe to monitor EMG and electrooculogram (EOG). The rats were habituated to the restraining and recording system for 8–10 days. At the end of the training, they could stay calm for 5–6 h daily sessions with a normal sleep-wake cycle. Two days before the first pharmacological session, under pentobarbital and local lidocaine anesthesia, a 4 mm hole was drilled over the vlPAG/dDpMe region to remove the dura under microscopic control. At the beginning of each recording session, the brain surface was cleaned under local lidocaine anesthesia.

#### Pharmacological protocol

Daily recording sessions were typically performed over a maximum of 7–10 days, each session lasting ∼4–6 hr. Drugs (Mus 10 mM, pH 4, Sigma; NaCl 0,9%; Phaseolus *vulgaris leucoagglutinin*, PHA-L, 1%, Vector Laboratories or Fluorogold, Fg, 4%) were applied iontophoretically during the light-on sleep period (between 10 a.m. and 1 p.m.) with a multi-barrel micropipette (20 µm tip diameter) lowered into the vlPAG/dDpMe region. Once the pipette was in place, and after a 15 min baseline recording, Mus or saline was ejected iontophoretically for 20 min at 100 nA. After the end of this injection, sleep-waking cycle was recorded for 1 h. Mus or saline (20 min, 100 nA) was injected in the same site at least 2 h after the end of the first recording. For each rat, one injection of tracer (PHA-L, Fg) was applied in the first Mus-positive injection site to localize it (Figure S5). At the end of the last injection, the rats were sacrificed under anesthesia and perfused with a fixative solution as described above.

#### Data analysis

W, SWS and PS were determined throughout the experiment on 10 s epochs by visual inspection of the EMG, EEG and the EEG power spectrum. Hypnograms were then drawn using a homemade script in Spike-2 software (CED). Each recording was decomposed on 4 consecutive 20 min epochs after the beginning of the Mus or saline injection. The effect latency was defined as the time-interval between the onset of drug application and the appearance of the first PS episode. The duration of the effect was defined as the time-interval between the effect onset and the moment at which the mean PS quantity value returned to the baseline value. The EEG spectral power was calculated with a Spike-2 script computing a fast Fourier transform on 10 s EEG epochs (0.1 Hz resolution). The EEG spectrogram was obtained using the ‘specgram.m’ function of Matlab (Math-Works, Natick, Massachusetts, USA). For this purpose, a sliding window Fourier transform was applied to the EEG signal using a 1 s window with a 0.25 s overlap. The mean±SEM of the vigilance states quantities were calculated for the effect of Mus and compared with those observed with saline injections using ANOVA followed by *post hoc* Bonferroni tests.

## Supporting Information

Figure S1 Schematic distribution of Fos+ (small black dots) and Fos-GAD (large red dots) neurons on coronal sections taken at 600 µm intervals from −5.60 and −6.80 from Bregma in a representative animal for PSC (left hand side), PSD (middle) and PSR (right hand side) conditions after Fos immunohistochemistry combined with GAD67 mRNA in situ hybridization. Abbreviations: 3, oculomotor nucleus; cp, cerebral peduncle, basal part; dlPAG, dorsolateral periaqueductal gray; dmPAG, dorsomedial periaqueductal gray; dDpMe, dorsal part of the deep mesencephalic nucleus; DpMe, deep mesencephalic nucleus; IP, interpeduncular nucleus; lPAG, lateral periaqueductal gray; ml, medial lemniscus; mlf, medial longitudinal fasciculus; PaR, pararubral nucleus; R, red nucleus; RRF, retrorubral field; SNC, substantia nigra, compact part; SNR, substantia nigra, reticular part; vlPAG, ventrolateral periaqueductal gray; VTA, ventral tegmental area.
(9.56 MB TIF)

Figure S2 Schematic distribution of Fos+ (small black dots) and Fos-GAD (large red dots) neurons on coronal sections taken at 600 µm intervals from −7.40 to −8.60 from Bregma in a representative animal for PSC (left hand side), PSD (middle) and PSR (right hand side) conditions after Fos immunohistochemistry combined with GAD67 mRNA in situ hybridization. Abbreviations: 4, trochlear nucleus; CGPn, central gray of the pons; CnF, cuneiform nucleus; cp, cerebral peduncle, basal part; DLL, dorsal nucleus of the lateral lemniscus; dlPAG, dorsolateral periaqueductal gray; dmPAG, dorsomedial periaqueductal gray; dDpMe, dorsal part of the deep mesencephalic nucleus; DpMe, deep mesencephalic nucleus; DRN, dorsal raphe nucleus; DTg, dorsal tegmental nucleus; IP, interpeduncular nucleus; LDTg, laterodorsal tegmental nucleus; lPAG, lateral periaqueductal gray; LPB, lateral parabrachial nucleus; me5, mesencephalic trigeminal tract; ml, medial lemniscus; mlf, medial longitudinal fasciculus; MnR, median raphe nucleus; Pn, pontine nuclei; PnO, pontine reticular nucleus, oral part; PPTg, pedunculopontine tegmental nucleus; py, pyramidal tract; RR, retrorubral nucleus; scp, superior cerebellar peduncle; SLD, sublaterodorsal nucleus; vlPAG, ventrolateral periaqueductal gray; VLL, ventral nucleus of the lateral lemniscus; VTg, ventral tegmental nucleus.
(10.14 MB TIF)

Figure S3 Schematic distribution of Fos+ (small black dots) and Fos-GAD (large red dots) neurons on coronal sections taken at 600 µm intervals from −9.20 to −10.40 from Bregma in a representative animal for PSC (left hand side), PSD (middle) and PSR (right hand side) conditions after Fos immunohistochemistry combined with GAD67 mRNA in situ hybridization. Abbreviations: 6, abducens nucleus; 7, facial nucleus; 7n, facial nerve; CGPn, central gray of the pons; CnF, cuneiform nucleus; g7, genu of the facial nerve; Gi, gigantocellular reticular nucleus; GiA, gigantocellular reticular nucleus, alpha part; icp, inferior cerebellar peduncle; KF, Kölliker-Fuse nucleus; LC, locus coeruleus; LDTg, laterodorsal tegmental nucleus; LPB, lateral parabrachial nucleus; LPGi, lateral paragigantocellular nucleus; me5, mesencephalic trigeminal tract; mlf, medial longitudinal fasciculus; Mo5, motor trigeminal nucleus; MVe, medial vestibular nucleus; PCRt, parvicellular reticular nucleus; Pn, pontine nuclei; PnC, pontine reticular nucleus, caudal part; PnV, pontine reticular nucleus, ventral part; Pr, prepositus nucleus; py, pyramidal tract; RMg, raphe magnus nucleus; RPa, raphe pallidus nucleus; scp, superior cerebellar peduncle; SLD, sublaterodorsal nucleus; SO, superior paraolivary nucleus; Sp5O, spinal trigeminal nucleus, oral part; sp5, spinal trigeminal tract.
(9.51 MB TIF)

Figure S4 Schematic distribution of Fos+ (small black dots) and Fos-GAD (large red dots) neurons on coronal sections taken at 600 µm intervals from −11.00 to −12.80 from Bregma in a representative animal for PSC (left hand side), PSD (middle) and PSR (right hand side) conditions after Fos immunohistochemistry combined with GAD67 mRNA in situ hybridization. Abbreviations: 7, facial nucleus; 12, hypoglossal nucleus; cDPGi, caudal part of the dorsal paragigantocellular nucleus; Gi, gigantocellular reticular nucleus; GiA, gigantocellular reticular nucleus, alpha part; GiV, gigantocellular reticular nucleus, ventral part; icp, inferior cerebellar peduncle; IO, inferior olive; LPGi, lateral paragigantocellular nucleus; mlf, medial longitudinal fasciculus; MVe, medial vestibular nucleus; PCRt, parvicellular reticular nucleus; Pr, prepositus nucleus; py, pyramidal tract; rDPGi, rostral part of the dorsal paragigantocellular nucleus; RMg, raphe magnus nucleus; ROb, raphe obscurus nucleus; RPa, raphe pallidus nucleus; Sol, nucleus of the solitary tract; Sp5O, spinal trigeminal nucleus, oral part; sp5, spinal trigeminal tract.
(9.31 MB TIF)

Figure S5 Photomicrograph illustrating a representative Phaseolus vulgaris leucoagglutinin (PHA-L) injection site in the vlPAG/dDpMe region used to localize muscimol and saline injections. Immunohistochemical detection of PHAL was made with the same sequential protocol described for Fos using a rabbit primary antibody to PHAL (1∶5000; DAKO, Denmark) and a DAB solution containing 0.6% of nickel ammonium sulphate. Abbreviations: 4, trochlear nucleus; Aq, Sylvius aqueduct; dDpMe, dorsal part of the deep mesencephalic nucleus; DpMe, deep mesencephalic nucleus;DRN, dorsal raphe nucleus; Me5, mesencephalic trigeminal nucleus; mlf, medial longitudinal fasciculus; vlPAG, ventrolateral periaqueductal gray. Scale bar: 500 µm.
(4.64 MB TIF)

Video S1 Video showing the behavior of a head-restrained rat and the simultaneous EOG, EMG and EEG recording during an episode of PS occurring after a Mus ejection in the vlPAG/dDpMe. The rat is immobile and display phasic movements of the vibrissae. The EOG shows the occurrence of REM, the EMG if flat and the EEG is activated and display theta activity.
(8.10 MB AVI)
