# Supplementary figures and images for: Localization of the Brainstem GABAergic Neurons Controlling Paradoxical (REM) Sleep
Source: PLoS One. 2009 Jan 26;4(1):e4272. doi: 10.1371/journal.pone.0004272 (PMC2629845; doi:10.1371/journal.pone.0004272)

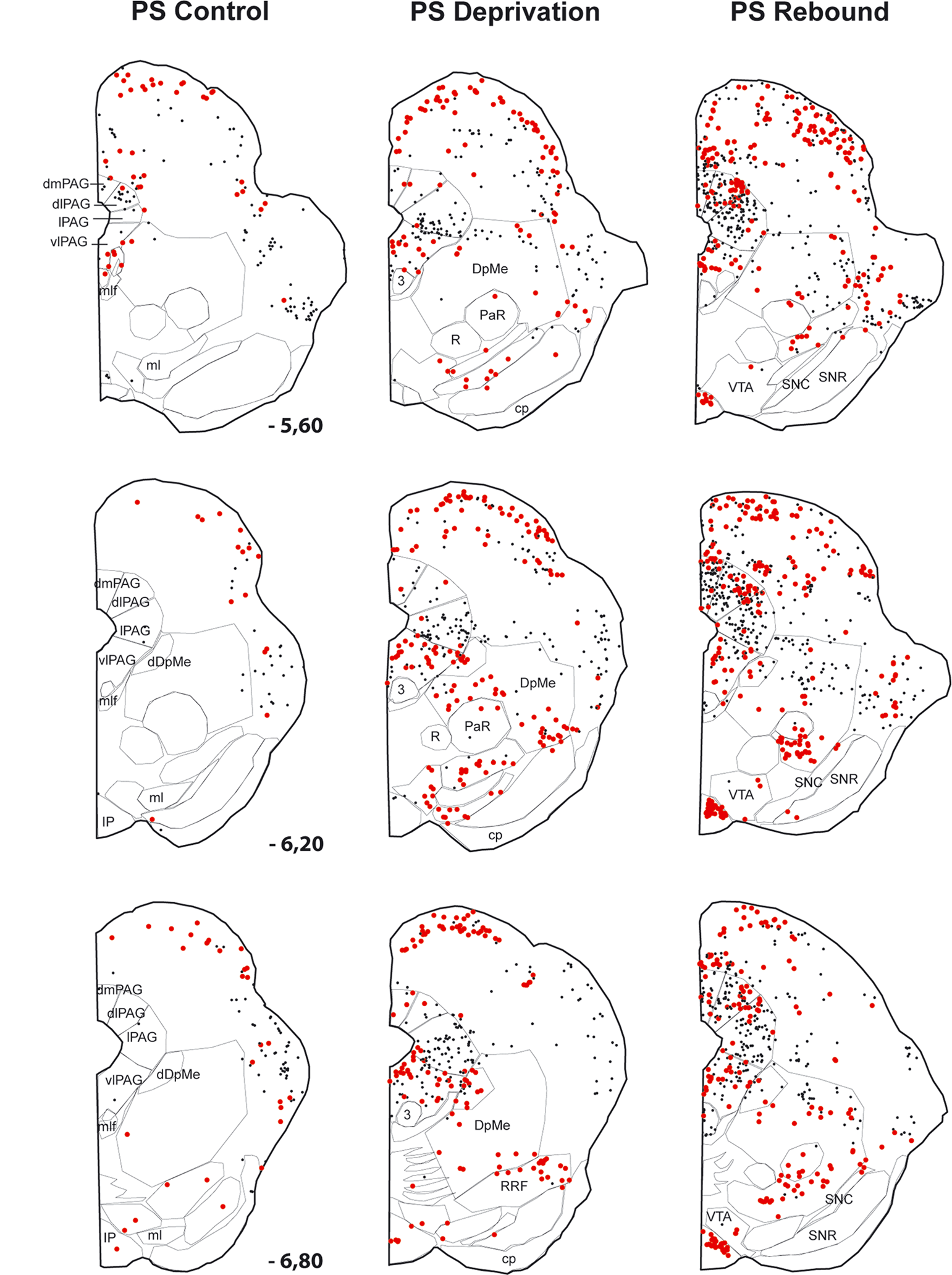

Supplement: Figure S1 — Schematic distribution of Fos+ (small black dots) and Fos-GAD (large red dots) neurons on coronal sections taken at 600 µm intervals from −5.60 and −6.80 from Bregma in a representative animal for PSC (left hand side), PSD (middle) and PSR (right hand side) conditions after Fos immunohistochemistry combined with GAD67 mRNA in situ hybridization. Abbreviations: 3, oculomotor nucleus; cp, cerebral peduncle, basal part; dlPAG, dorsolateral periaqueductal gray; dmPAG, dorsomedial periaqueductal gray; dDpMe, dorsal part of the deep mesencephalic nucleus; DpMe, deep mesencephalic nucleus; IP, interpeduncular nucleus; lPAG, lateral periaqueductal gray; ml, medial lemniscus; mlf, medial longitudinal fasciculus; PaR, pararubral nucleus; R, red nucleus; RRF, retrorubral field; SNC, substantia nigra, compact part; SNR, substantia nigra, reticular part; vlPAG, ventrolateral periaqueductal gray; VTA, ventral tegmental area. (9.56 MB TIF) [file pone.0004272.s001.tif]

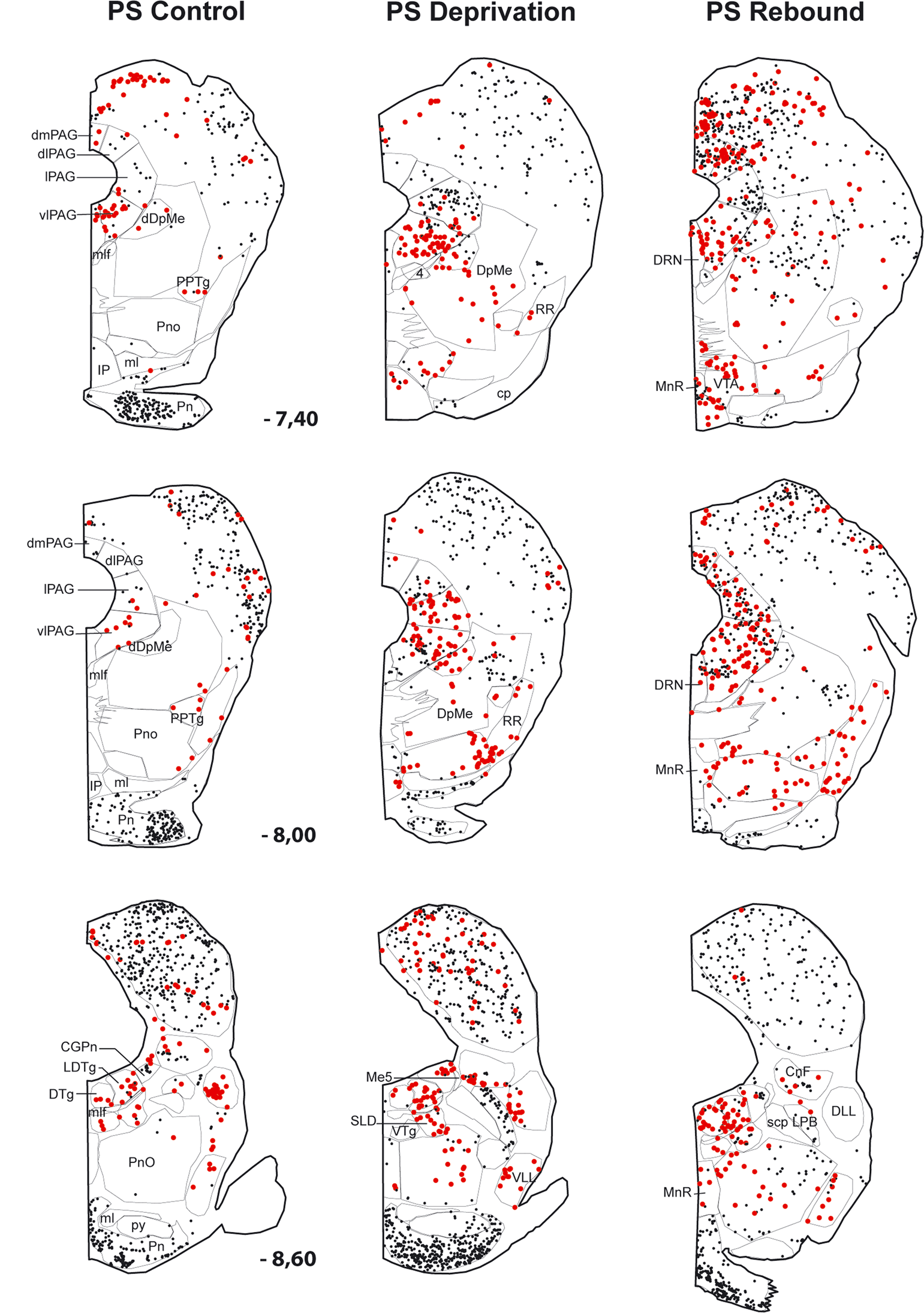

Supplement: Figure S2 — Schematic distribution of Fos+ (small black dots) and Fos-GAD (large red dots) neurons on coronal sections taken at 600 µm intervals from −7.40 to −8.60 from Bregma in a representative animal for PSC (left hand side), PSD (middle) and PSR (right hand side) conditions after Fos immunohistochemistry combined with GAD67 mRNA in situ hybridization. Abbreviations: 4, trochlear nucleus; CGPn, central gray of the pons; CnF, cuneiform nucleus; cp, cerebral peduncle, basal part; DLL, dorsal nucleus of the lateral lemniscus; dlPAG, dorsolateral periaqueductal gray; dmPAG, dorsomedial periaqueductal gray; dDpMe, dorsal part of the deep mesencephalic nucleus; DpMe, deep mesencephalic nucleus; DRN, dorsal raphe nucleus; DTg, dorsal tegmental nucleus; IP, interpeduncular nucleus; LDTg, laterodorsal tegmental nucleus; lPAG, lateral periaqueductal gray; LPB, lateral parabrachial nucleus; me5, mesencephalic trigeminal tract; ml, medial lemniscus; mlf, medial longitudinal fasciculus; MnR, median raphe nucleus; Pn, pontine nuclei; PnO, pontine reticular nucleus, oral part; PPTg, pedunculopontine tegmental nucleus; py, pyramidal tract; RR, retrorubral nucleus; scp, superior cerebellar peduncle; SLD, sublaterodorsal nucleus; vlPAG, ventrolateral periaqueductal gray; VLL, ventral nucleus of the lateral lemniscus; VTg, ventral tegmental nucleus. (10.14 MB TIF) [file pone.0004272.s002.tif]

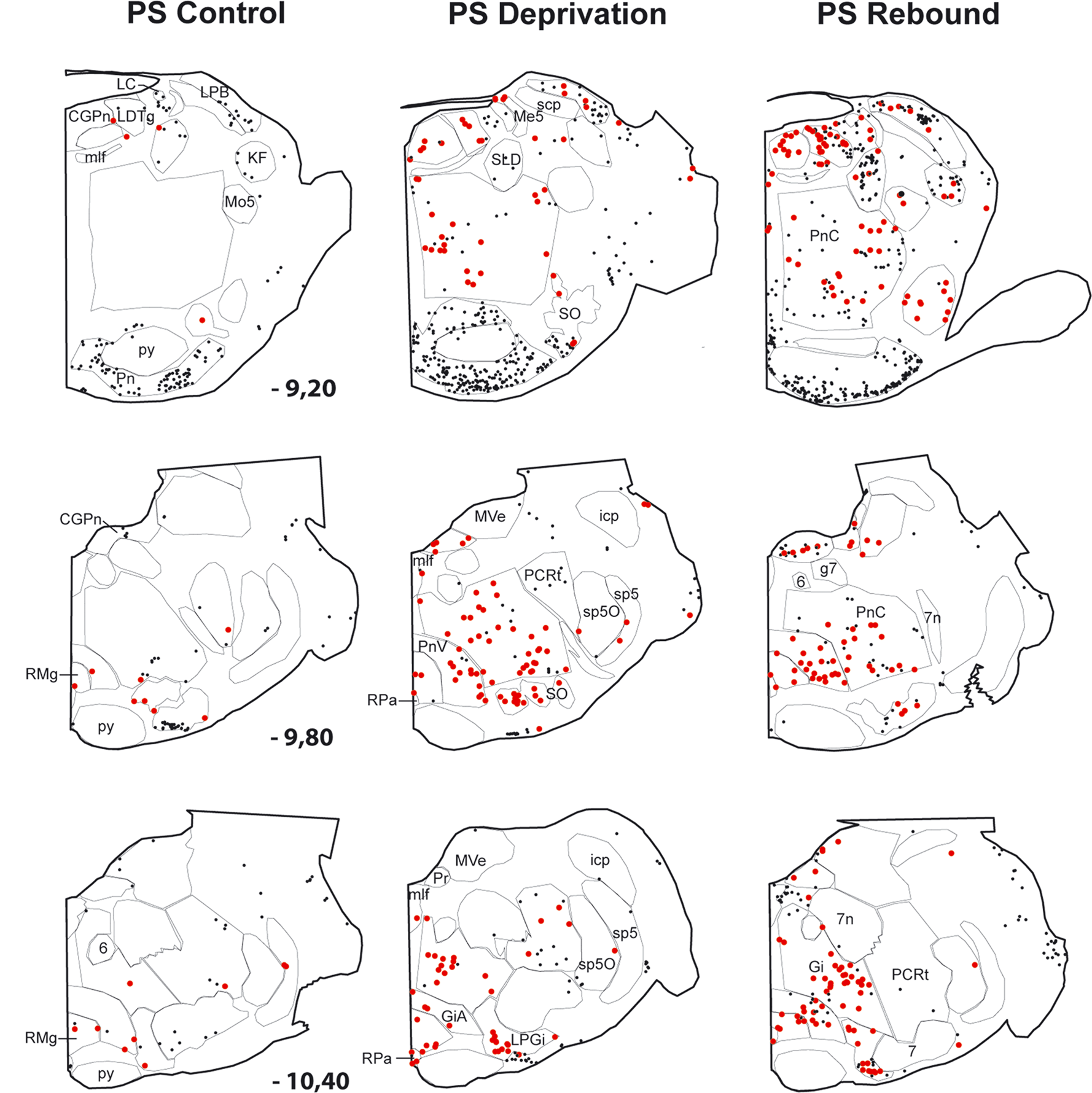

Supplement: Figure S3 — Schematic distribution of Fos+ (small black dots) and Fos-GAD (large red dots) neurons on coronal sections taken at 600 µm intervals from −9.20 to −10.40 from Bregma in a representative animal for PSC (left hand side), PSD (middle) and PSR (right hand side) conditions after Fos immunohistochemistry combined with GAD67 mRNA in situ hybridization. Abbreviations: 6, abducens nucleus; 7, facial nucleus; 7n, facial nerve; CGPn, central gray of the pons; CnF, cuneiform nucleus; g7, genu of the facial nerve; Gi, gigantocellular reticular nucleus; GiA, gigantocellular reticular nucleus, alpha part; icp, inferior cerebellar peduncle; KF, Kölliker-Fuse nucleus; LC, locus coeruleus; LDTg, laterodorsal tegmental nucleus; LPB, lateral parabrachial nucleus; LPGi, lateral paragigantocellular nucleus; me5, mesencephalic trigeminal tract; mlf, medial longitudinal fasciculus; Mo5, motor trigeminal nucleus; MVe, medial vestibular nucleus; PCRt, parvicellular reticular nucleus; Pn, pontine nuclei; PnC, pontine reticular nucleus, caudal part; PnV, pontine reticular nucleus, ventral part; Pr, prepositus nucleus; py, pyramidal tract; RMg, raphe magnus nucleus; RPa, raphe pallidus nucleus; scp, superior cerebellar peduncle; SLD, sublaterodorsal nucleus; SO, superior paraolivary nucleus; Sp5O, spinal trigeminal nucleus, oral part; sp5, spinal trigeminal tract. (9.51 MB TIF) [file pone.0004272.s003.tif]

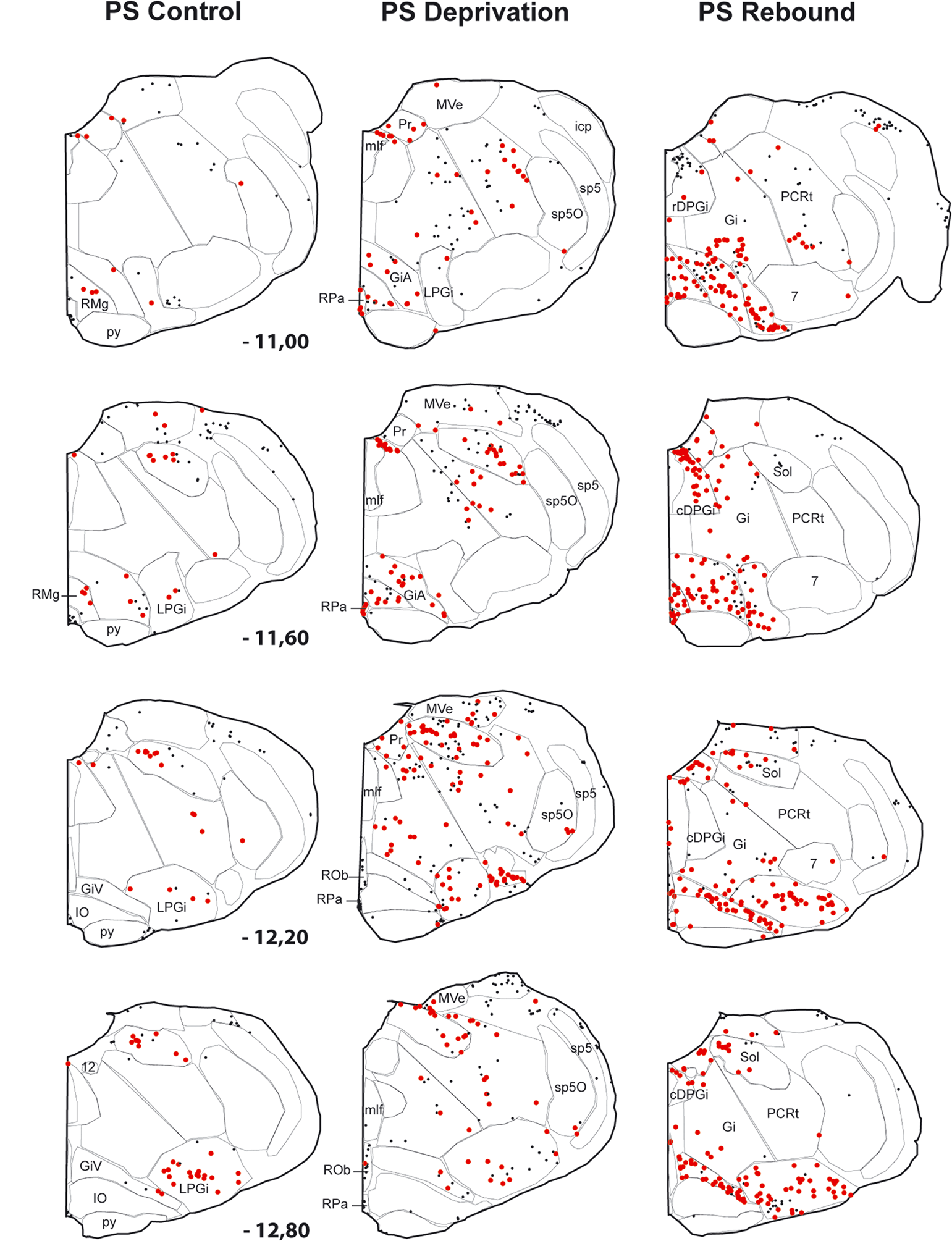

Supplement: Figure S4 — Schematic distribution of Fos+ (small black dots) and Fos-GAD (large red dots) neurons on coronal sections taken at 600 µm intervals from −11.00 to −12.80 from Bregma in a representative animal for PSC (left hand side), PSD (middle) and PSR (right hand side) conditions after Fos immunohistochemistry combined with GAD67 mRNA in situ hybridization. Abbreviations: 7, facial nucleus; 12, hypoglossal nucleus; cDPGi, caudal part of the dorsal paragigantocellular nucleus; Gi, gigantocellular reticular nucleus; GiA, gigantocellular reticular nucleus, alpha part; GiV, gigantocellular reticular nucleus, ventral part; icp, inferior cerebellar peduncle; IO, inferior olive; LPGi, lateral paragigantocellular nucleus; mlf, medial longitudinal fasciculus; MVe, medial vestibular nucleus; PCRt, parvicellular reticular nucleus; Pr, prepositus nucleus; py, pyramidal tract; rDPGi, rostral part of the dorsal paragigantocellular nucleus; RMg, raphe magnus nucleus; ROb, raphe obscurus nucleus; RPa, raphe pallidus nucleus; Sol, nucleus of the solitary tract; Sp5O, spinal trigeminal nucleus, oral part; sp5, spinal trigeminal tract. (9.31 MB TIF) [file pone.0004272.s004.tif]

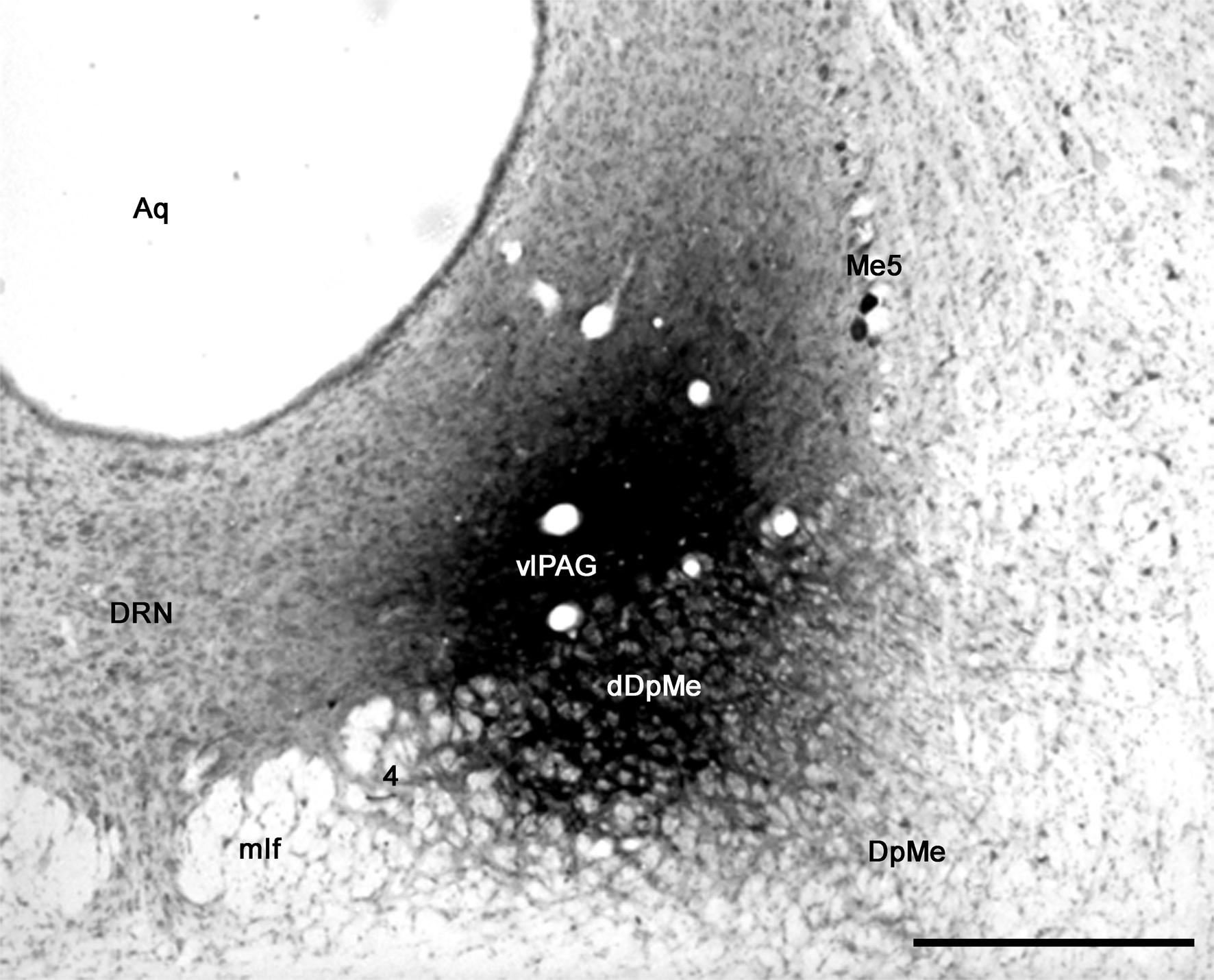

Supplement: Figure S5 — Photomicrograph illustrating a representative Phaseolus vulgaris leucoagglutinin (PHA-L) injection site in the vlPAG/dDpMe region used to localize muscimol and saline injections. Immunohistochemical detection of PHAL was made with the same sequential protocol described for Fos using a rabbit primary antibody to PHAL (1∶5000; DAKO, Denmark) and a DAB solution containing 0.6% of nickel ammonium sulphate. Abbreviations: 4, trochlear nucleus; Aq, Sylvius aqueduct; dDpMe, dorsal part of the deep mesencephalic nucleus; DpMe, deep mesencephalic nucleus;DRN, dorsal raphe nucleus; Me5, mesencephalic trigeminal nucleus; mlf, medial longitudinal fasciculus; vlPAG, ventrolateral periaqueductal gray. Scale bar: 500 µm. (4.64 MB TIF) [file pone.0004272.s005.tif]
